# Hemostatic therapies and ambiguous abnormal bleeding in cardiac operating rooms: A real-world, case-based evaluation of clinical practices

**DOI:** 10.1016/j.xjon.2026.101933

**Published:** 2026-06-18

**Authors:** Alexandre Sebestyen, Alexandre Behouche, Jean-Noël Evain, Julien Picard, Olivier Chavanon, Pierre Albaladejo

**Affiliations:** a Department of Cardiac Surgery, Grenoble Alpes University Hospital, Grenoble, France; b Alps Research Assessment and Simulation Centre, Grenoble Alpes University, Grenoble, France; c Department of Anesthesia and Intensive Care, Grenoble Alpes University Hospital, Grenoble, France

**Keywords:** cardiac surgery, intraoperative hemorrhage, case-based simulation, situational awareness, patient blood management

## Abstract

**Objective:**

Hemostatic therapy is commonly used in cardiac surgery. Despite tools to reduce inappropriate use, variability remains, mainly due to a lack of standards, especially intraoperatively. This study examined practices in cardiac operating rooms using a standardized clinical case.

**Methods:**

French cardiac anesthesiologists and surgeons responded to a nationwide questionnaire, including a multiple-choice test based on a clinical case of ambiguous bleeding despite normal tests. The use of hemostatic therapies during surgery—reversal, chest closure, and transfer—was assessed with a fictitious surgeon or anesthesiologist involved in the assessment. Interrater reliability was measured using Gwet's AC1, and ratings before and after the intervention were compared with a paired Fisher exact test. Nonclinical factors and perceptions of bleeding were evaluated using a Likert scale.

**Results:**

Interrater reliability was poor among 73 anesthesiologists (γ = 0.38 [95% confidence interval, 0.03 to 0.73]) and 31 surgeons (γ = 0.16 [95% confidence interval, –0.31 to 0.63]). Users' rates significantly increased since chest closure, mainly after the fictitious colleague intervention, regardless of profession (from 29.0% to 89.3% [*P* < .0001] among surgeons, and from 34.2% to 52.1% [*P* = .0446] among anesthesiologists). Surgeons reported lower satisfaction and a greater tendency to judge transfusions as insufficient or delayed, possibly due to the greater influence of transfusion policy. There was a trend toward divergence in clotting characteristics ratings with intraoperative bleeding.

**Conclusions:**

Hemostatic therapy may require standardization to better manage intraoperative bleeding. Teamwork, especially with surgeons, is vital for shared awareness in these complex situations.

## Graphical Abstract

**Figure:**
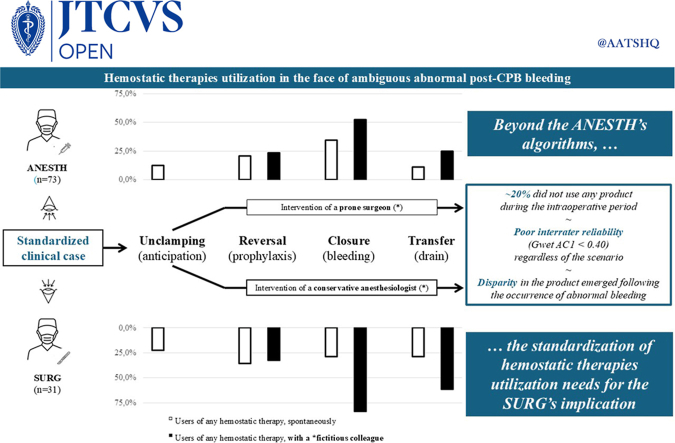

**Figure:**
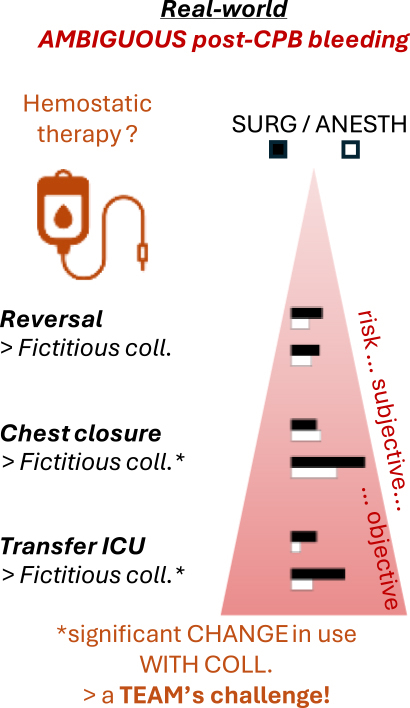
Standardized case-based simulation of ambiguous bleeding to assess hemostatic therapy.

**Central Message**

Inconsistencies in hemostatic therapy show a lack of standard in the operating room, surgeons might influence hemostatic therapy use, and shared awareness of bleeding could enhance its management.

**Perspective**

Clinical case-based complex situations help evaluate cardiac anesthesiologists and surgeons in decision making and awareness. Postcardiopulmonary bypass ambiguous bleeding, common in practice, could be an effective model. Simulations might train physicians to enhance their nontechnical skills.

Bleeding management is a cornerstone of patient safety in surgery.^1^ In cardiac surgery, bleeding is particularly challenging because cardiopulmonary bypass (CPB) induces specific biologic disturbances, including hemodilution, consumption of coagulation factors, and platelet dysfunction.^2^ Accordingly, hemostatic therapies play a central role in the hemostatic armamentarium.^3^ Clinical practice guidelines generally guide perioperative hemostatic therapies utilization. However, the intraoperative setting remains poorly supported by evidence, resulting in substantial practice variability. Within the framework of Patient Blood Management (PBM),^4^^,^^5^ as advocated by the World Health Organization, the utilization of hemostatic therapies has remained relatively overlooked, despite evidence linking inappropriate transfusion practices to increased morbidity.^6^^,^^7^

With the growing implementation of point-of-care viscoelastic testing, algorithm-based decision making is now recommended to reduce inappropriate use.^4^ Although viscoelastic testing has significantly reduced hemostatic therapies utilization,^8^ its overall influence remains modest, and practice variability persists.^9^ These limitations are partly explained by the major influence of human factors,^10^ especially hospital culture,^11^^,^^12^ but also by the clinician's individual experience when managing bleeding situations.^13^^,^^14^ Aside from overtly life-threatening bleeding, in which liberal transfusion may be lifesaving, any transfusion administered after reversal therapy—which is relatively common in daily practice—places cardiac surgeons and anesthesiologists in a complex clinical situation.^15^^,^^16^ In such scenarios, a high level of situational awareness is required to support optimal decision making and management.^17^^,^^18^

In a prior study, we explored the interrater reliability in the judgment of decontextualized bleeding situations.^16^ This decontextualization allowed us to study the perception (level 1 of situational awareness) of anesthesiologists and surgeons in the face of different post-CPB bleeding, thereby distinguishing ambiguous situations from clearly normal or abnormal ones. However, this decontextualization limited the inference of residual coagulopathy and the anticipation of severe bleeding that required the use of hemostatic therapy upfront. Because uncertainties may lead to errors in practice, the objective of this observational study was to analyze individual hemostatic therapy utilization in operating rooms among cardiac surgeons and anesthesiologists managing a selected ambiguous abnormal bleeding situation in a recontextualized real-world clinical case.

## Method

### Ethical Statement

The study protocol was reviewed and approved by the Clinical Research and Innovation Department of Grenoble-Alpes University Hospital and by the Scientific Committee of the French Society of Anesthesiology and Intensive Care (IRB No. 00010254 – 2025 – 104) on July 4, 2025. Completion of the questionnaire implied the respondent's informed consent to participate in the study and to the publication of the study data, and no personally identifiable information was collected.

### Questionnaire

The questionnaire was divided into 3 parts as follows:

**Part 1: Respondent characteristics and center features.** First, respondents provided information on their professional profile, including years in practice (<10 or ≥10 years), adult cardiac surgery activity (exclusive or nonexclusive, individual case volume), training background in cardiac surgery (training at 2 or more centers, training abroad), and years of service at their current center (<10 or ≥10 years). They also provided general information about their center, including institutional affiliation and center volume, as well as specific characteristics related to bleeding management, such as blood bank location, availability of viscoelastic testing and a decision-making algorithm, and the presence of a PBM program.

**Part 2. Real-world clinical case**. First, respondents were then shown a real-world clinical case selected from our local database of post-CPB video clips, in which intraoperative bleeding had been arbitrarily classified as ambiguously abnormal.^16^ Hemostatic laboratory parameters were within the normal range. The intraoperative period was divided into 3 phases for decision making: at the time of reversal therapy, during chest closure, and before transfer to the intensive care unit. Clinical and nonclinical data were provided progressively, with scenarios adapted to the respondent's specialty. Respondents were asked to choose among several hemostatic management options, when applicable: additional biologic testing, heparin neutralization (protamine readministration), blood-derived hemostatic therapy (among platelet concentrates, fresh frozen plasma, fibrinogen concentrate, prothrombin complex concentrate, recombinant activated factor VII), surgical reexploration, or no intervention. At each step, respondents answered twice: first spontaneously, and then after input from a fictitious colleague—either a transfusion-conservative anesthesiologist for surgeon respondents or a transfusion-prone surgeon for anesthesiologist respondents. At the time of aortic unclamping, respondents were also asked whether they would anticipate ordering hemostatic blood components—platelet concentrates and fresh frozen plasma. Appendix E1 provides an English translation of each scenario.

**Part 3: Assessment of human factors**. We also explored nonclinical factors in hemostatic therapy management. Using 5-point Likert scales, respondents were asked to rate their satisfaction with both the appropriateness and the timing of their hemostatic therapy utilization in this clinical case, as well as their perception of the potential influence of selected nonclinical factors on their transfusion practices in general. With respect to the clinical case, a video clip showing “ambiguously abnormal” bleeding, as arbitrarily defined by local experts at our center,^16^ was shown during reassessment before chest closure (Video 1). The clip was then replayed, and respondents were asked, again using 5-point Likert scales, to rate their perception of this intraoperative bleeding according to 4 cardinal characteristics: flow, location, color, and clot formation.

### Questionnaire Distribution and Data Management

The questionnaire was distributed to all cardiac surgeons and cardiothoracic anesthetists registered by the French Society of Thoracic and Cardiovascular Surgery and the Heart-Thorax-Vessels Anesthesiology Section of the French Society of Anesthesiology and Intensive Care. Study data were collected and managed using the secure web-based software platform Research Electronic Data Capture (Vanderbilt University).

### Statistical Analysis

Only fully completed questionnaires were included in the analysis. Respondent characteristics and center features were summarized as counts and percentages and compared between surgeons and anesthesiologists using Fisher exact test with Bonferroni correction when appropriate. According to the scenario, we analyzed the proportion of respondents who, among the different solutions, administered any hemostatic therapy prophylactically at reversal therapy and therapeutically during chest closure and before transfer to the intensive care unit. First, interrater reliability was assessed using Gwet's AC1 coefficient (γ) with 95% CI. Reliability was classified as excellent (γ > 0.80), good (γ = 0.60-0.80), moderate (γ = 0.40-0.60), or poor (γ < 0.40). Second, at each step, response rates before and after input from the fictitious colleague were compared using a McNemar exact test. For hemostatic blood components, we calculated the proportion of respondents who anticipated transfusion by ordering these products at the time of aortic unclamping. Finally, the human factors assessment was summarized as counts (percentages) or median (interquartile range [IQR]) and compared between surgeons and anesthesiologists using Fisher exact test with a Bonferroni correction when appropriate or the Kruskal-Wallis test. All analyses were performed using R software (R Foundation for Statistical Computing).

## Results

### Respondent Characteristics and Center Features

The questionnaires were distributed to 346 surgeons (all in the cardiac adult specialty) and 818 anesthesiologists (some in the cardiac adult specialty) registered with their respective societies. One hundred fifty-one physicians opened the questionnaires: 42 surgeons, 88 anesthesiologists, and 21 with an undefined specialty. We analyzed 104 fully completed questionnaires, including 31 from surgeons and 73 from anesthesiologists, representing 43 of the 63 cardiac surgery centers in France (see Table 1).

**Table 1:** Respondents’ characteristics and center features

Characteristics	Overall	Anesthesiologists	Surgeons	*P* value∗
	(n = 104)	(n = 73)	(n = 31)	
Characteristic				
Length of expertise >10 y	54 (51.9)	35 (47.9)	19 (61.3)	.2838
Adult cardiac surgery only	26 (25.0)	6 (21.9)	20 (64.5)	**<.0001**
Training from 2+ centers	55 (52.9)	29 (39.7)	26 (83.9)	**<.0001**
Training abroad	18 (17.3)	2 (2.7)	16 (51.6)	**<.0001**
Individual volume activity				.0236
<100/y	22 (21.2)	20 (27.4)	2 (6.5)	–
100-200/y	50 (48.1)	30 (41.1)	20 (64.5)	–
>200/y	32 (30.8)	23 (31.5)	9 (29.0)	–
Work in several centers	4 (3.8)	2 (2.7)	2 (6.5)	.5807
Length of work in the center >10 y	50 (48.1)	28 (38.4)	22 (71.0)	**.0028**
Salaried practice	86 (82.7)	62 (84.9)	24 (77.4)	.4001
Center feature				
Public affiliation	80 (76.9)	59 (80.8)	21 (67.7)	.2027
Center volume activity				.2523
<500/y	16 (15.4)	14 (19.2)	2 (6.5)	–
500-1000/y	67 (64.4)	44 (60.3)	23 (74.2)	–
>1000/y	21 (20.2)	15 (20.5)	6 (19.4)	–
Blood bank location				.3172
In the same building	27 (27.0)	21 (28.8)	6 (19.4)	–
In the same center	53 (51.0)	38 (52.1)	15 (48.4)	–
Outside the center	24 (23.1)	14 (19.2)	10 (32.3)	–
Point-of-care viscoelastic test	89 (85.6)	63 (86.3)	26 (89.3)	.7653
Transfusion algorithm	75 (72.1)	55 (75.3)	20 (64.5)	.3392
PBM program	97 (93.3)	67 (91.8)	30 (96.8)	.6712

Values are presented as median (range) or n (%).*PBM*, Patient Blood Management.

∗ For a categorical variable with k >2 values, Bonferroni's correction for the *P* value was applied, then a 2-by-2 comparison was done for each value if applicable. Boldface type in the *P* value column indicates a significant difference.

Cardiac surgeons were more likely than anesthesiologists to work exclusively in adult cardiac surgery (64.5% vs 21.9%; *P* < .0001), to have trained at multiple cardiac surgery centers (83.9% vs 39.7%; *P* < .0001), to have completed training abroad (51.6% vs 2.7%; *P* < .0001), and to have worked at their current center for more than 10 years (71.0% vs 38.4%; *P* = .0028). No significant between-group differences were observed in center characteristics.

### Hemostatic Therapies Utilization in Cardiac Operating Rooms

**Figure 1:**
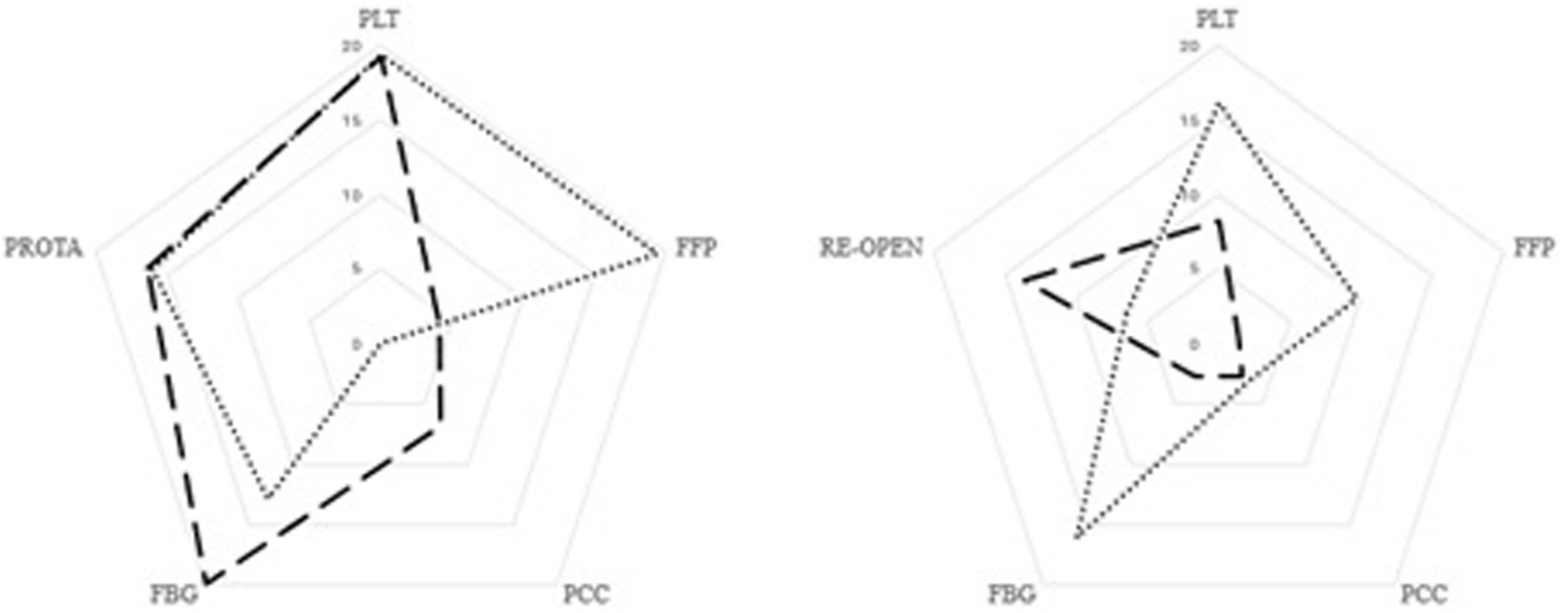
Abnormal bleeding management. During chest closure (*left*), anesthesiologists (*interrupted line*) and surgeons (*interrupted dot*) used platelet concentrates (*PLT*) similarly with protamine reinfusion. In contrast, anesthesiologists preferred fibrinogen concentrate (*FBG*), and surgeons preferred fresh frozen plasma (*FFP*). Before transfer (*right*), anesthesiologists preferred reopening, whereas surgeons preferred PLT, FFP, and FBG transfusions. No respondents used recombinant activated factor VII.

The distribution of spontaneous responses to intraoperative bleeding is shown in Figure 1. During chest closure, 93.5% of surgeons and 84.5% of anesthesiologists recommended surgical reexploration. Platelet concentrates were used more frequently than protamine readministration by both surgeons and anesthesiologists. Surgeons more often favored fresh frozen plasma, whereas anesthesiologists more often favored fibrinogen concentrate. Before transfer to the intensive care unit, 64.5% of surgeons and 64.4% of anesthesiologists recommended repeat laboratory testing. Compared with protamine readministration, anesthesiologists more often favored reopening for surgical exploration, whereas surgeons more often favored platelet concentrates and fibrinogen concentrate.

An alluvial diagram illustrating the utilization of hemostatic therapies, from reversal therapy to transfer to the intensive care unit, is presented in Appendix E2. About 16.5% of anesthesiologists and 39.3% of surgeons did not use any product throughout the clinical case. Most physicians who administered hemostatic therapy early continued transfusion once abnormal bleeding developed. We noted higher disorganization in hemostatic therapy utilization when bleeding occurred, and the fictitious colleague appeared to be a critical point associated with marked dispersion in the utilization of hemostatic therapies. Although surgeons widely used labile blood products, anesthesiologists seemed to prefer fibrinogen and showed greater variation in the types of blood products.

**Figure 2:**
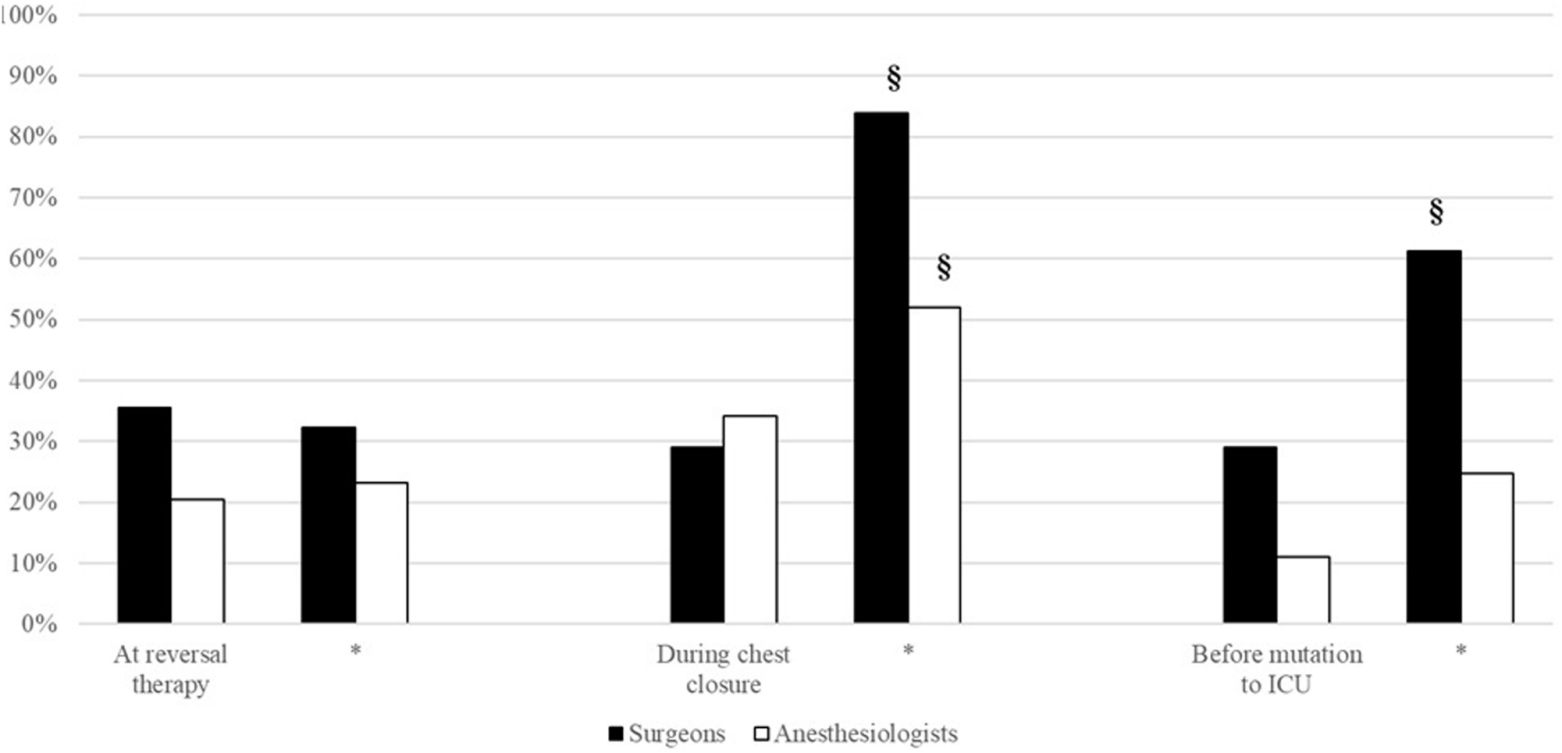
Rate of hemostatic therapy users among surgeons and anesthesiologists during the intraoperative period at each step. There was a significant increase (§) of users with the fictitious colleague's point of view (∗) for both surgeons and anesthesiologists, at chest closure and at transfer for surgeons only. *ICU*, Intensive care unit.

Interrater reliability was poor regardless of scenario, with Gwet's AC1 coefficients of 0.38 (IQR, 0.03 to 0.73) among anesthesiologists and 0.16 (IQR, −0.31 to 0.63] among surgeons. The rate of hemostatic therapy users increased significantly after the intervention of the fictitious colleague among both surgeons and anesthesiologists, at chest closure (from 29.0% to 89.3%; *P* < .0001, and from 34.2% to 52.1%; *P* = .0146, respectively) and before transfer to the intensive care unit (from 29.0% to 61.3%; *P* = .0063, and from 11.0% to 24.7%; *P* = .0265, respectively), although these rates did not significantly differ at aortic unclamping. By contrast, no significant differences were observed in reversal therapy (from 35.5% to 32.2%; *P* = 1 among surgeons, and from 20.5% to 23.3%; *P* = .8417 among anesthesiologists) (Figure 2).

At aortic unclamping, 9 anesthesiologists (12.3%) and 7 surgeons (22.6%) anticipated the need for hemostatic blood components. Ultimately, 25 anesthesiologists (34.2%; 1 wasted unit) transfused the clinical case spontaneously, and 36 (49.3%; 1 wasted unit) did so after input from the fictitious transfusion-prone surgeon. Among surgeons, 13 (41.9%; no wasted units) transfused spontaneously, and 23 (74.2%; no wasted units) did so after input from the fictitious transfusion-conservative anesthesiologist.

### Self-Assessment, Nonclinical Factors, and Perception of Bleeding

**Figure 3:**
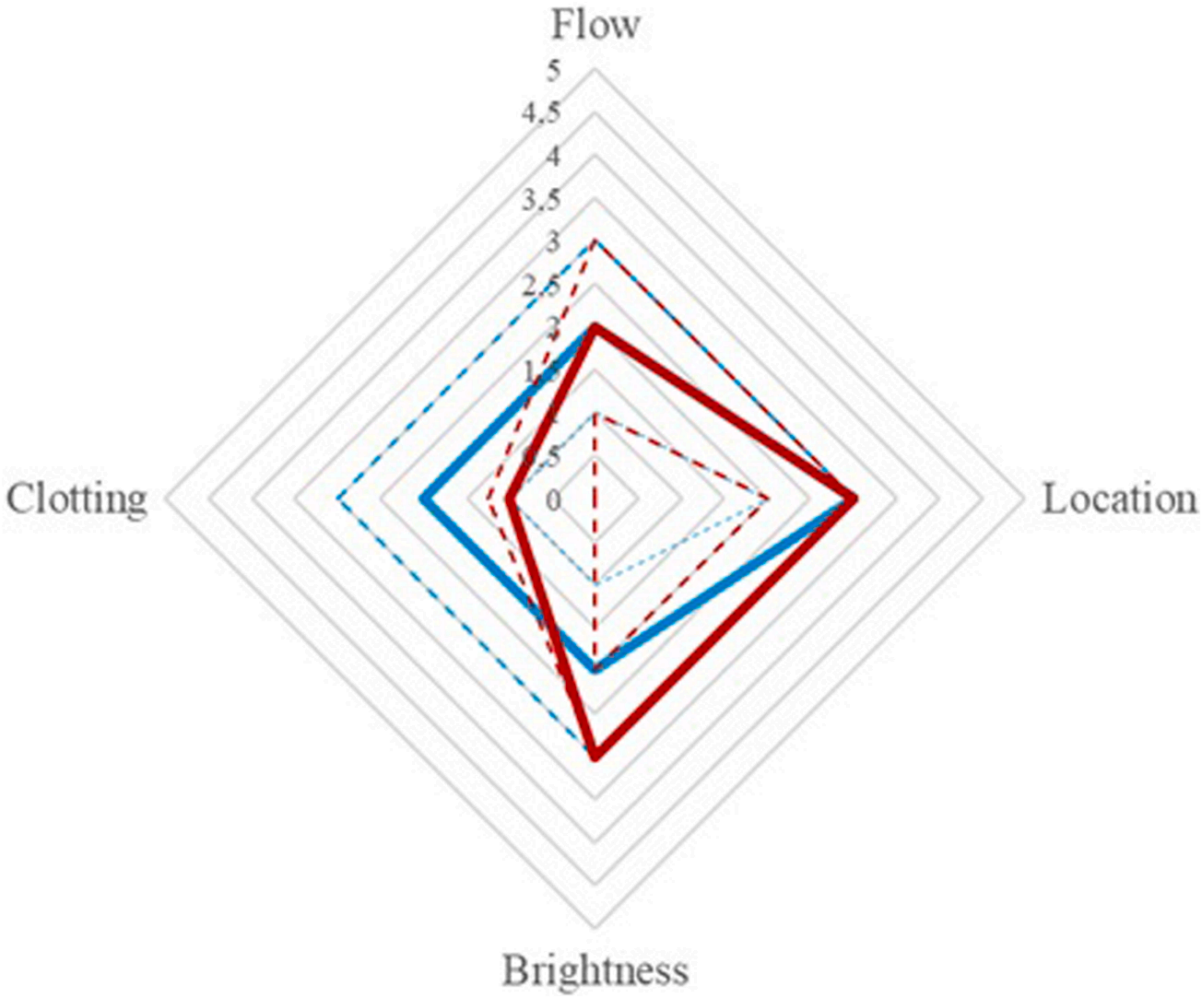
Perception of the intraoperative bleeding based on cardinal characteristics. Although surgeons (*red line*) and anesthesiologists (*blue line*) similarly noted the flow and location, there was a trend in differences in brightness and clotting characteristics between physicians.

Surgeons reported lower satisfaction than anesthesiologists with their management of the case (3 [IQR, 1.5-3] vs 4 [IQR, 2-4]; *P* = .0088), with a greater tendency to judge transfusion as insufficient or delayed (Table 2). In routine practice, most respondents considered inappropriate prophylactic utilization to be associated with consequences, either clinical or economic. They expressed a perceived need for greater standardization of practice across specialties. Regarding nonclinical factors, surgeons reported less influence from resource availability (2 [IQR, 2-3.5] vs 3 [IQR, 2-4]; *P* = .0208) but tended to report greater influence from transfusion policy (3 [IQR, 2-3] vs 2 [IQR, 1-3]; *P* = .0694). Whereas ratings of bleeding flow and location were similar between anesthesiologists and surgeons, there was a trend toward divergence in ratings for the clotting feature (2 [IQR, 1-3] vs 1 [IQR, 0-1.25]; *P* = .1086) (Figure 3).

**Table 2:** Exploration of human factors on individual practices

Characteristics	Overall (N = 104)	Anesthesiologists (n = 73)	Surgeons (n = 31)	*P* value∗
Autoevaluation				
Satisfaction with the clinical case	3 (2-4)	3 (2-4)	2 (1.5-3)	**.0088**
Feelings about utilization in the clinical case				**<.0001**
Insufficient	5 (4.8)	1 (1.4)	4 (12.9)	**.0268**
Delayed	13 (12.5)	3 (4.1)	10 (32.3)	**.0002**
Anticipative	9 (8.7)	9 (12.3)	0	.0547
Excessive	5 (4.8)	3 (4.1)	2 (6.5)	.6331
Main impact of inappropriate prophylactic utilization				.6916
Clinical	50 (48.1)	34 (46.6)	16 (51.6)	–
Economical	42 (40.4)	31 (42.7)	11 (35.5)	–
Ethical	10 (9.6)	6 (8.2)	4 (12.9)	–
Need for standardization in general	95 (91.3)	66 (90.4)	29 (93.5)	.7215
Nonclinical factors in bleeding management				
Work commitments	0 (0-1)	0 (0-1)	0 (0-1)	.0998
Personal commitments	0 (0-0)	0 (0-0)	0 (0-0)	.9097
Available resources	3 (2-4)	3 (2-4)	2 (2-3.5)	**.0208**
Transfusion policy	2 (1-3)	2 (1-3)	3 (2-3)	.0694
Day/hour of procedure	1 (0-2)	1 (0-2)	0 (0-2)	.1482
Colleague's personality in the operating room	2 (1-2.25)	2 (1-3)	1 (1-2)	.4181
Colleague's judgment on bleeding/transfusion	2 (1-3)	2 (1-3)	2 (1-3)	.9793
Perception (/5) of bleeding (video clip) on cardinal features				
Flow (0: no/…/5: life-threatening)	2 (1-3)	2 (1-3)	2 (1-3)	.4488
Location (0: diffuse/…/5: local)	3 (2-3)	3 (2-3)	3 (2-3)	.2821
Color/red (0: clear/…/5: dark)	2 (1.75-3)	2 (1-3)	3 (2-3)	.1517
Clotting (0: fluid/…/5: firm)	2 (1-3)	2 (1-3)	1 (0-1.25)	.1086

Values are presented as median (range) or n (%).

∗ For a categorical variable with k >2 values, Bonferroni's correction for the *P* value was applied, then a 2-by-2 comparison was done for each value. Boldface type in the *P* value column indicates a significant difference.

## Discussion

Simulation tools are adapted to evaluate practice under nonstandard conditions. The present study completes our prior research on variability in judgment across different bleeding situations.^16^ This prior work validated across a nationwide study our arbitrary classification of ambiguously abnormal bleeding, from which we selected 1 real-world clinical case with some friction points. This real-world clinical case-based exploration of individual practice in the utilization of post-CPB hemostatic therapies reveals substantial variation in decision making under intraoperative uncertainty.

First, the poor interrater reliability observed throughout the intraoperative period highlights the low level of standardization of practice in the cardiac operating room, which remains an ongoing public health issue. Although the proportions of hemostatic therapy users among surgeons and anesthesiologists were relatively similar in the initial spontaneous responses, we observed a significant increase beginning at chest closure—when abnormal bleeding occurred—particularly after input from the fictitious colleague, regardless of the scenario. This finding illustrates 2 major points: perception of abnormal bleeding appeared to depend more on the colleague's interpretation than on the clinical bleeding risk factors or even the respondent's own initial perception; and the surgeon, whether real or fictitious, seemed to exert a strong influence on hemostatic management decisions. In an asymmetrical scenario, with a transfusion-prone fictitious surgeon versus a conservative-transfusion fictitious anesthesiologist, we expected convergence in responses regarding the use of hemostatic therapies, with increases by anesthesiologists and decreases by surgeons, respectively. The unexpected increase by surgeons suggested imposing their belief in residual coagulopathy despite the anesthesiologist's judgment. Besides, we note in the alluvial diagram that some anesthesiologists change their hemostatic therapy strategy after the fictitious surgeon's intervention, whereas all the surgeons who spontaneously used hemostatic therapy continued to do so after the fictitious anesthesiologist's intervention. Second, administration of reversal therapy, despite the absence of any evidence-based indication in this clinical case, demonstrated the persistence of prophylactic utilization. Conversely, the contrast between the low rate of advance ordering of hemostatic blood components and the eventual need for their administration suggests a limited ability to anticipate abnormal bleeding situations based on clinical risk factors alone, with a consequent risk of delayed transfusion. Although respondents were generally satisfied with their practice, surgeons more frequently perceived transfusion as delayed, not because of resource limitations, but rather because of transfusion policy.

For the past 3 decades, it has been well recognized that transfusion practice in cardiac surgery varies widely.^19^, ^20^, ^21^ Although packed red blood cell transfusion has long been studied, leading to the development of PBM strategies, hemostatic therapies deserve the same level of attention.^22^ Indeed, several authors have reported an association between high-transfusion centers and worse morbidity and mortality in cardiac surgery.^6^^,^^7^ Beyond the clinical influence, which remains difficult to interpret because of substantial bias, inappropriate transfusion undoubtedly increases costs, thereby supporting the use of test-guided algorithms.^4^ Hemostatic therapies are administered more frequently during the intraoperative period.^3^^,^^6^^,^^14^^,^^23^ Aside from a few prophylactic indications (eg, platelet count <50 G/L) or exceptional life-threatening bleeding requiring massive transfusion, hemostatic therapies are justified primarily to correct coagulopathy associated with abnormal bleeding. The introduction of point-of-care viscoelastic testing and the resulting reduction or redistribution have demonstrated our limited ability to predict coagulopathy based solely on clinical bleeding risk factors.^8^ Nevertheless, these tests are not sufficient, and substantial variability in practice persists.^9^

Beyond center differences, individual practice also varies considerably,^13^^,^^14^ largely as a result of hospital culture.^24^^,^^25^ The alluvial diagram illustrated this variability, predominantly when the bleeding occurred. Intraoperative bleeding follows a form of fuzzy logic, and ambiguously abnormal bleeding is relatively common in daily practice once blood becomes visible in the operative field,^16^ placing physicians in complex clinical situations.^15^ In the face of profound uncertainty, optimizing operational decision making requires a high level of situational awareness.^17^^,^^18^ Applying Endsley's framework,^26^ optimal transfusion management requires anticipation (level 3) of major bleeding—defined retrospectively by its clinical consequences—caused by coagulopathy, after first understanding it (level 2) and perceiving it (level 1), as ultimately structured in decision-making algorithms. In our study, although biologic test results were pending or remained within the normal range, hemostatic therapy users paradoxically increased regardless of the scenario, especially within the real surgeon/fictitious transfusion-conservative anesthesiologist dyad.

Biologic testing has been developed to guide hemostatic therapy by improving the understanding of coagulopathy. However, its interpretation still depends heavily on the perception of surgical bleeding, which remains essential for translating test results into transfusion action, as emphasized in all decision-making algorithms. Typically defined as excessive, microvascular, and diffuse bleeding,^27^ this perception remains subjective.^28^ By exploring perceptions of intraoperative bleeding across four cardinal parameters, the median ratings in our study retrospectively confirmed the ambiguous nature of the bleeding. Although anesthesiologists and surgeons rated flow and location similarly—that is, whether the bleeding was excessive and diffuse—they tended to diverge in their assessment of clotting. Yet, in particular, clotting is related to biologic findings, especially viscoelastic properties measured by point-of-care testing.

In the setting of such inconsistency, the colleague's perception seems essential in validating the abnormality of the bleeding, more so than clinical predictive factors alone. By virtue of their role, surgeons have the greatest experience with the operative field, as reflected in our cohort by their higher individual case volume and more frequent training at multiple centers or abroad. If practice is to move toward greater standardization, surgeons ought to be fully engaged in decision making,^29^^,^^30^ provided that their training extends beyond mentorship alone, which is itself shaped by both individual experience and prior hospital culture. Such full involvement might also improve surgeons' satisfaction by reducing their perception of delayed therapy.

Closure checklists and the 5-minute test have been proposed to support the assessment of surgical bleeding by providing information on macrovascular sources and on flow and diffusion, respectively, thereby reducing reexploration and perioperative transfusion.^31^, ^32^, ^33^, ^34^ More recently, the Validated Intraoperative Bleeding scale has made this information available at a glance.^35^ By using them, surgeons slow down the course of the procedure and take a dedicated time to manage bleeding. Although these tools individually reduced reexploration and transfusion, combining them would be more interesting.^36^ By promoting a shared mental model of bleeding, this “surgical” characterization might help anesthesiologists interpret biologic test results, and could be easily implemented in a decision-making algorithm.^37^^,^^38^ However, it remains poorly suited to capturing the specific nature of post-CPB coagulopathy. In addition to reinforcing the need to adapt evidence when evidence is limited, decision making algorithms may also help structure communication about bleeding between the 2 protagonists in the operating theater, finally, as a script.

### Strengths, Limitations, and Perspectives

To our knowledge, this is the first exploration of transfusion practice based on a real-life, progressive clinical case. This study may pave the way for the use of similarly simple tools to evaluate physicians’ situational awareness in complex clinical situations. Although the subdivision of the intraoperative period may seem somewhat artificial, it enabled us to explore the dynamic nature of intraoperative decision making, which would not have been possible with a single preoperative label, as previously proposed.^11^

The objective of this study was to explore individual practice, regardless of the actual patient outcome. To validate the uncertainty of this ambiguous situation, no hemostatic therapy was administered intraoperatively or within the first 48 postoperative hours in the real case, and the patient did not experience any major bleeding event as defined by the Universal Definition of Perioperative Bleeding, such as reexploration or transfusion of more than 1 U packed red blood cells during the hospital stay.^39^ In the absence of an established standard of care against which to compare strategies, we cannot—and do not—claim that our management approach is superior to alternative approaches. Once a standard is established, strict concordance testing to evaluate practice will be conducted with validated experts. Without a gold standard, we noted a high rate of physicians using any hemostatic therapy—about half of anesthesiologists and three-quarters of surgeons—despite the absence of biological evidence of residual coagulopathy. This wide divergence from the real-world case confirmed the need to explore each level of situational awareness—perception, comprehension, and anticipation—up to the decision, to reveal and correct errors in practice. In the last part of the questionnaire, we noted that perceptions of bleeding and feelings in clinical practice were not entirely similar between anesthesiologists and surgeons, paving the way for future research.

The main limitation of this study is the relatively small number of respondents, likely because completing the questionnaire in full required approximately 15 minutes, which is probably too long.^38^ The reliability among surgeons was not significantly certain, probably because the number of respondents was too small, but was probably insufficient because the major part of the CI of Gwet’s AC1 was under 0.60. Besides, the small number of surgeons led to an overestimate of the variation in respondents’ rates. The respondents' rate was about 9% for surgeons and 14.6% (considering the mean number of anesthesiologists by center was 8).

This work was conducted to hypothesize about shared situational awareness rather than to analyze it. We explored only individual responses, without examining teamwork and the sharing process within a dyad of respondents. Real-time clinical case-based simulation, with a real or a fictitious and adaptive colleague, would be a more attractive approach for exploring and even training physicians in teamwork and, ultimately, in shared situational awareness for optimal operational decision making. The development of a common tool for surgeons and anesthesiologists to jointly manage post-CPB bleeding—similar to the Validated Intraoperative Bleeding scale but incorporating coagulopathy and clotting parameters—could be of interest in future practice to improve overall PBM in cardiac surgery.

## Conclusions

Post-CPB hemostatic therapy utilization remains variable, and, in the context of PBM, the appropriate use of hemostatic therapies should be the rule, rather than restrictive use of packed red blood cells during the perioperative period. Despite the development of several tools to guide bleeding management, the complexity of the intraoperative bleeding might contribute to this ongoing public health issue. Sharing the same mental model of abnormal bleeding would be key. In addition to specific tools for standardized analysis and communication, clinical-case-based evaluation and training could be useful for educating both cardiac surgeons and anesthesiologists to improve their required cognitive and social nontechnical skills.

### Declaration of Generative AI and AI-Assisted Technologies in the Writing Process

During the preparation of this work, the authors used ChatGPT-5 (Open AI) to assist with translation and language refinement. The authors reviewed and edited the content as needed and take full responsibility for the publication's content.

## Conflict of Interest Statement

The authors reported no conflicts of interest.

The *Journal* policy requires editors and reviewers to disclose conflicts of interest and to decline handling or reviewing manuscripts for which they may have a conflict of interest. The editors and reviewers of this article have no conflicts of interest.

## Figures and Tables

**Figure:**
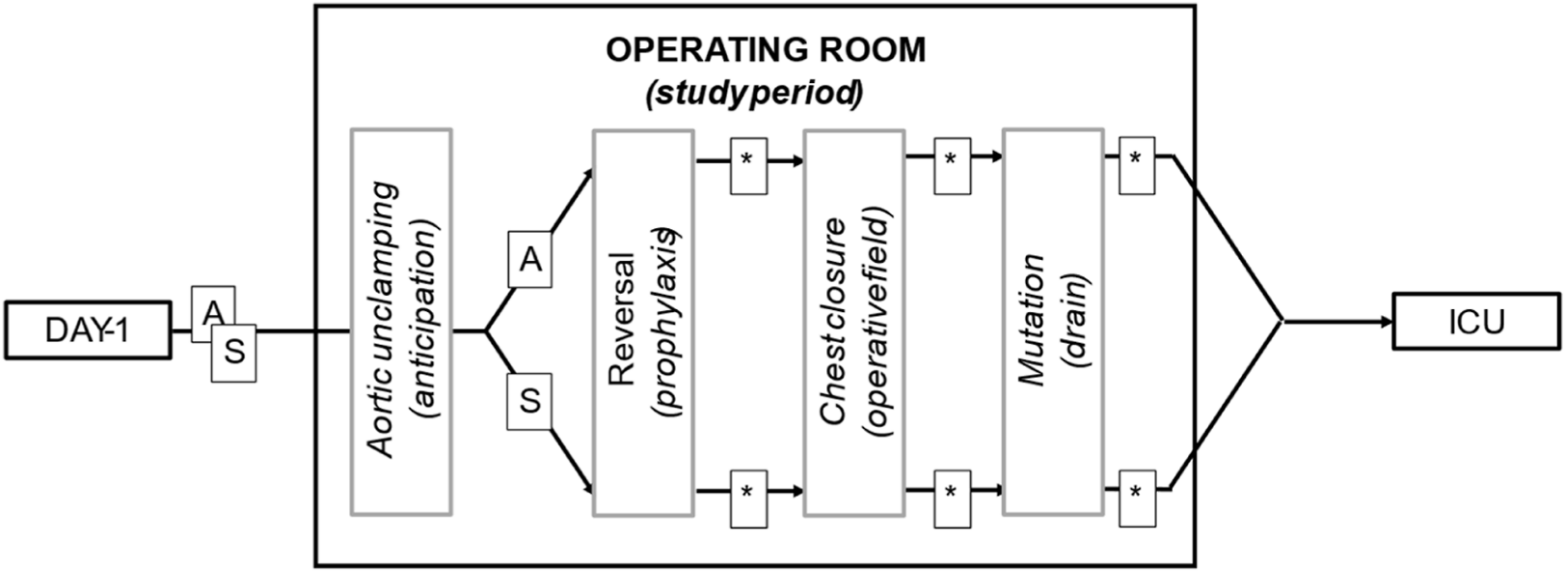


**Table:** 

	The videoclip of the bleeding is available following the link: https://vimeo.com/1076310450

**Figure:**
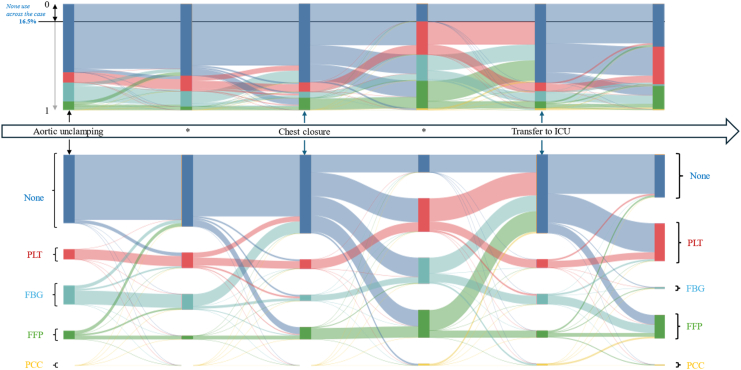


**Figure:**
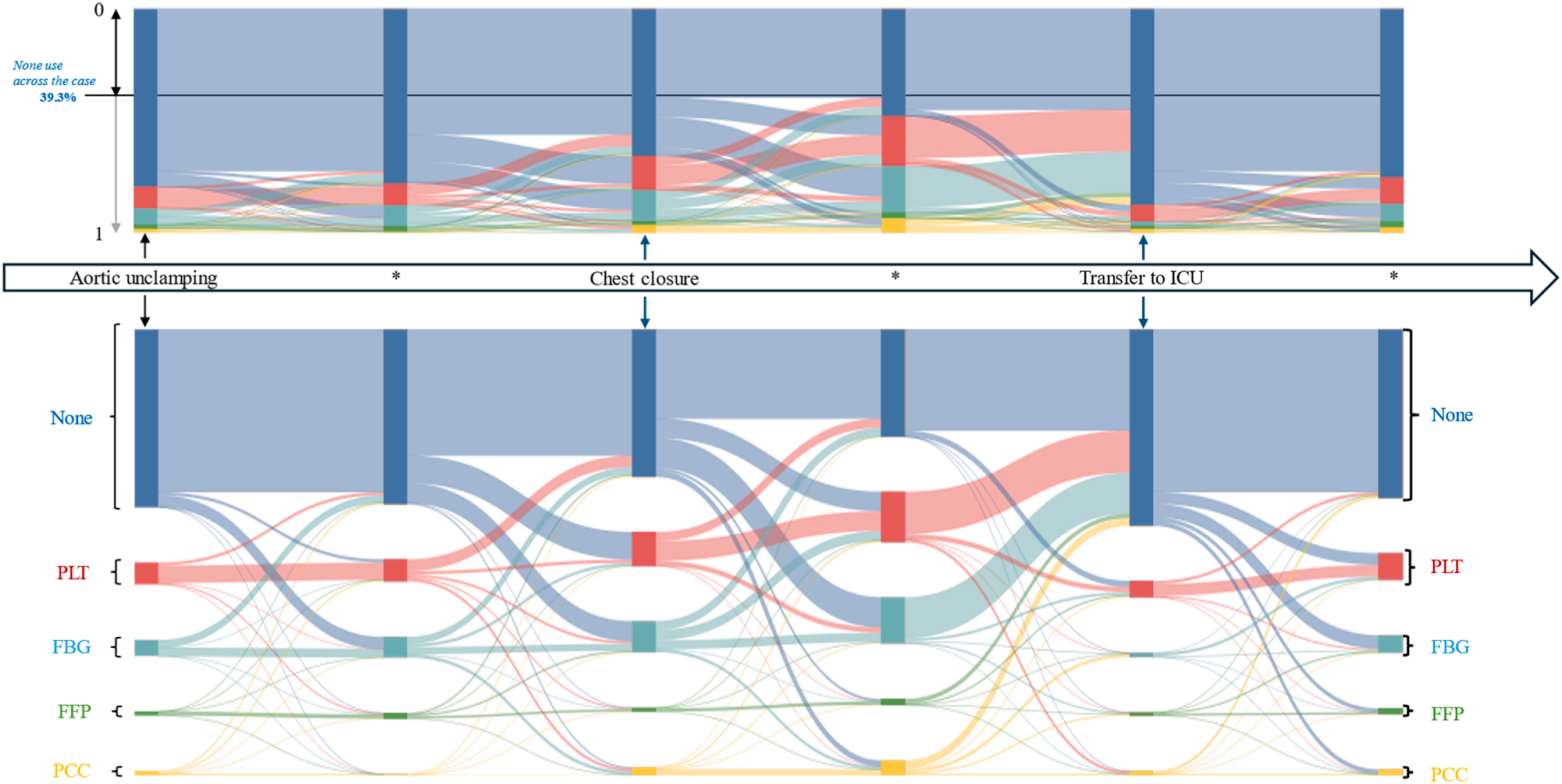


## Supplementary Data

Video 1 For the chest closure step of the clinical case, we provided a video clip of postcardiopulmonary bypass bleeding recorded 10 minutes after reversal therapy, assuming that anticipated coagulopathy is treated. This video was stored in a local database (https://vimeo.com/1076310450), for which we arbitrarily defined the bleeding situation as ambiguous. This definition was validated in a prior nationwide study, in which interrater reliability was poor and significantly lower than in both clearly normal and clearly abnormal situations. Video available at: https://www.jtcvs.org/article/S2666-2736(26)00356-6/fulltext.

## Appendix E1. Standardized Clinical Case

The standardized clinical case below is fictitious; any resemblance to real persons (or actual events) is purely coincidental. Cardiac anesthetists and surgeons were required to read the context thoroughly to fulfill their respective roles, and they subsequently answered a multiple-choice test.

For comprehension, the clinical case was illustrated below. During operating room steps, anesthetists and surgeons were faced with a respective scenario with a fictitious colleague (∗) regarding respondents' profession, a pro-transfusion surgeon for anesthetists and a cons-transfusion anesthetist for surgeons, respectively, and they were answered twice, first spontaneously and then after the fictitious colleague's intervention. At step 5 (intensive care unit), they answered directly after a fictitious intensivist who noted the bleeding.

Below is a scheme of the progressive clinical case:

Each anesthetist (A) and surgeon (S) responded at 6 progressive steps. Although the day before the surgery (Day 1) and the intensive care unit (ICU) steps were identical, the operating room scenario was adapted for each specialty, with the intervention of a fictitious colleague (∗) at 4 decision points in hemostasis and transfusion.

### Clinical Case: Preoperative Label

Miss A. is a 67-year-old woman, 68 kg/1.65 m, hospitalized for planned surgical aortic valve replacement with coronary artery bypass grafting, for exertional dyspnea.

#### Medical history

- High blood pressure
- Noninsulin-requiring type 2 diabetes mellitus
- Weaned tobacco intoxication
- Coronary stenting
- Nonembolic permanent atrial fibrillation
- Asymptomatic peripheral arterial disease
- Rheumatoid arthritis
- Appendicitis during childhood
- No prior major bleeding event
- No allergy

#### Usual treatment

- Enalapril 20 mg once daily, pended for 1 day
- Metformine 1000 mg once daily, pended for 2 days
- Acetylsalicylic acid 160 mg once daily
- Apixaban 5 mg twice daily, pended for 4 days without relay
- Methotrexate 25 mg weekly
- Folic acid 10 mg weekly

#### Preoperative work-up

- Electrocardiogram: atrial fibrillation 80 to 90 bpm, electric left ventricular hypertrophy
- Chest X-ray: subnormal
- Coronarography: 3-vessel disease with 50% to 70% stenose on the distal common left trunk on right dominance. Right coronary artery occluded
- Transthoracic echocardiogram: severe aortic stenosis without other heart valve disease; normal ventricular function (left ventricular ejection fraction 65% without segmental hypo/dyskinesis, tricuspid annular plane systolic excursion 18 mm); Ventricular hypertrophy and dilated left atrium; no aortic dilatation; no pericardial effusion.
- Doppler: stenosis of the right superficial femoral artery with a systolic pressure index of 0.67; stenosis 70% of the right carotid
- Biology assessment: Group O — hemoglobin 13 g/dL, transferrin saturation coefficient 21%, ferritin 180 ng/mL — platelet 134 G/L, prothrombin time 87%, activated partial thromboplastin time 1.1, fibrinogen 2.9 g/L — C- reactive protein 7.2 mg/L — creatinine 160 μmol/L, liver enzymes < 3N, thyroid-stimulating hormone in normal range.

### Step 1: Aortic Unclamping

Miss A. is undergoing quadruple coronary artery bypass grafting with bilateral internal thoracic artery—the left 1 on left anterior descending artery and the right 1 on ramus intermedius through passing through transverse sinuses and 2 saphenous grafts on obtuse marginal and the posterior descending artery separately, aortic valve replacement with a 23-mm bioprosthesis, and left atrial appendage was cut-and-sew. Rewarming is in progress. The aorta is unclamped at 126 min.

You are on duty tonight.

**What labile hemostatic blood product do you order?**

☐ Platelet concentrate
☐ Fresh frozen plasma
☐ None of the above

### Step 2: Reversal Therapy

Cardiopulmonary bypass was stopped at 187 minutes, with arterial pressure controlled just under 0.25 μg/kg/minute norepinephrine. The cardiopulmonary system was weaned, and reversal therapy with protamine sulfate was started.

**What additional hemostatic blood components do you propose upfront?**

☐ Platelet concentrate
☐ Fresh frozen plasma
☐ Prothrombin complex concentrate
☐ Fibrinogen concentrate/cryoprecipitate
☐ Recombinant activated factor VII
☐ None of the above

*∗ Surgeon scenario*: The anesthetist tells you that protamine sulfate will be sufficient.

*∗ Anesthetist scenario*: The surgeon tells you that protamine sulfate will not be sufficient.

**What do you propose, finally, upfront?**

☐ Platelet concentrate
☐ Fresh frozen plasma
☐ Prothrombin complex concentrate
☐ Fibrinogen concentrate/cryoprecipitate
☐ Recombinant activated factor VII
☐ None of the above

### Step 3: Chest Closure

Reversal therapy was infused for 10 minutes. Temperature is 36.3 °C. You observe the operative site at a glance before chest closure. Biological test is pending…

**What do you propose to do faced with this bleeding in the field?**

☐ To administer protamine again
☐ To transfuse platelet concentrate
☐ To transfuse fresh frozen plasma
☐ To administer prothrombin complex concentrate
☐ To administer fibrinogen concentrate/cryoprecipitate
☐ To administer recombinant activated factor VII
☐ To reexplore the operative field
☐ None of the above

*∗ Surgeon scenario:* The anesthetist tells you it's bleeding and it's not medical.

*∗ Anesthetist scenario*: The surgeon tells you it's bleeding and it's not surgical.

3000 IU protamine is reinfused.

**What do you propose, finally?**

☐ To transfuse platelet concentrate
☐ To transfuse fresh frozen plasma
☐ To administer prothrombin complex concentrate
☐ To administer fibrinogen concentrate/cryoprecipitate
☐ Recombinant activated factor VII
☐ To reexplore the operative field
☐ None of the above

### Step 4: Transfer to the Intensive Care Unit

The sternotomy is closed, and the skin is oozing slightly. Rhythm is 90 bpm sinus, and arterial pressure is stabilized <0.38 μg/kg/minute norepinephrine. Temperature is 36.1 °C. Drains are not dry; dark fluid is present, mainly from the retrosternal drain. The canister was at 50 mL, just enough time to place the dressing and remove the drapes.

The biological test is normal.

**What do you propose to do in the face of this bleeding through the drains?**

☐ New biological test
☐ To transfuse platelet concentrate
☐ To transfuse fresh frozen plasma
☐ To administer prothrombin complex concentrate
☐ To administer fibrinogen concentrate/cryoprecipitate
☐ To administer recombinant activated factor VII
☐ To reopen the sternotomy
☐ None of the above

*∗ Surgeon scenario:* The anesthetist tells you it's bleeding and it's not medical.

*∗ Anesthetist scenario*: The surgeon tells you it's bleeding and it's not surgical.

Blood is taken for a new test.

**What do you propose, finally?**

☐ To transfuse platelet concentrate
☐ To transfuse fresh frozen plasma
☐ To administer prothrombin complex concentrate
☐ To administer fibrinogen concentrate/cryoprecipitate
☐ Recombinant activated factor VII
☐ To reopen the sternotomy
☐ None of the above

## Appendix E2. Alluvial Flowchart of Utilization of Hemostatic Therapies

Below are the alluvial flowcharts of the respondents' use of hemostatic therapies across the clinical case.

The flowcharts are shown twice: compacted (above) to measure the rate of respondents who did not use any product across the case and exploded (below) to show changes in decision by product type.

Above is the alluvial flowchart of surgeons’ use of hemostatic therapies: 16.5% did not use any products throughout the clinical case. In the exploded view (below), we found that they preferred to use platelet concentrate (PLT) and fresh frozen plasma (FFP) after the transfusion-conservative fictitious anesthesiologist intervened, since bleeding occurred, and until the transfer to the intensive care unit.

The alluvial flowchart above shows anesthesiologists’ use of hemostatic therapies: 39.3% did not use any products throughout the clinical case. In the exploded view (below), we found that they preferred to use PLT and fibrinogen (FBG) after the transfusion-prone fictitious surgeon intervened, since bleeding occurred. Compared with surgeons’ responses, we observed greater change in blood product utilization, even though some respondents did not maintain their spontaneous decisions after the fictitious surgeons' intervention. *ICU*, Intensive care unit; *PCC*, prothrombin complex concentrate.
